# Local administration of submicron particle paclitaxel to solid carcinomas induces direct cytotoxicity and immune-mediated tumoricidal effects without local or systemic toxicity: preclinical and clinical studies

**DOI:** 10.1007/s13346-020-00868-4

**Published:** 2020-11-06

**Authors:** Shelagh Verco, Holly Maulhardt, Michael Baltezor, Emily Williams, Marc Iacobucci, Alison Wendt, James Verco, Alyson Marin, Sam Campbell, Paul Dorman, Gere diZerega

**Affiliations:** 1 US Biotest, Inc. 231 Bonetti Drive, Suite 240, San Luis Obispo, CA 93401, USA; 2 CritiTech Particle Engineering Solutions, LLC, 1849 E 1450 Road, Lawrence, KS 66044, USA; 3 NanOlogy, LLC, 3909 Hulen Street, Fort Worth, TX 76107, USA; 4 DFB Pharmaceuticals, Inc, 3909 Hulen Street, Fort Worth, TX 76107, USA

**Keywords:** NanoDoce, NanoPac, Ovarian cancer, Pancreatic cancer, Pancreatic cysts, Prostate cancer, Lung cancer

## Abstract

This report describes local administration of submicron particle paclitaxel (SPP) (NanoPac®: ~ 800-nm-sized particles with high relative surface area with each particle containing ~ 2 billion molecules of paclitaxel) in preclinical models and clinical trials evaluating treatment of carcinomas. Paclitaxel is active in the treatment of epithelial solid tumors including ovarian, peritoneal, pancreatic, breast, esophageal, prostate, and non-small cell lung cancer. SPP has been delivered directly to solid tumors, where the particles are retained and continuously release the drug, exposing primary tumors to high, therapeutic levels of paclitaxel for several weeks. As a result, tumor cell death shifts from primarily apoptosis to both apoptosis and necroptosis. Direct local tumoricidal effects of paclitaxel, as well as stimulation of innate and adaptive immune responses, contribute to antineoplastic effects. Local administration of SPP may facilitate tumor response to systemically administered chemotherapy, targeted therapy, or immunotherapy without contributing to systemic toxicity. Results of preclinical and clinical investigations described here suggest that local administration of SPP achieves clinical benefit with negligible toxicity and may complement standard treatments for metastatic disease.

**Graphical abstract:**

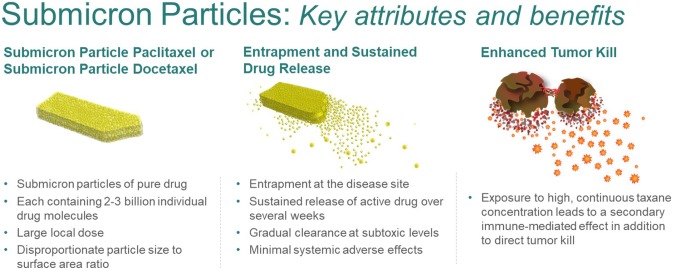

## Introduction

Paclitaxel is a high molecular weight, highly protein bound, hydrophobic molecule, which limits its access to tumor cells. IV administration of paclitaxel achieves relatively low levels of drug at the tumor for a short period of time, resulting in intermittent tumor cell exposure to drug resulting in death that is typically apoptotic and may allow for some tumor cell survival, division, and/or mutation potentially resulting in paclitaxel resistance.

Local treatment of solid tumors by systemic therapies has the potential to increase tumoricidal effects without increasing systemic toxicity. It has long been hypothesized that local, sustained tumor treatment would make the drug available to tumor cells over multiple cell-division cycles, resulting in tumoricidal benefits without compromising the patient’s quality of life [1]. Various particle-based drug delivery systems are being developed for treatment of cancer [2, 3]. However, success of many systems is limited by ineffective delivery to tumor sites as well as abbreviated drug residence due, in part, to the clearance of particles by the immune system [4].

In order to address the limitations of IV treatment modalities, submicron particles of paclitaxel (SPP, NanoPac®, NanOlogy, Ft Worth, TX), with a mean particle size of ~ 800 nm (containing approximately 2 billion paclitaxel molecules each) with a high surface area to facilitate molecule release, were developed to allow for suspension in an aqueous media [5]. These aqueous suspensions of SPP allow for various routes of local administration including inhalation (IH) via nebulizer, intraperitoneal (IP) delivery, or intratumoral (IT) injection.

This review will describe the therapeutic potential of SPP for treatment of various solid tumors and associated clinical trials, all of which demonstrate clinical utility in small Phase 1/2 studies (Table 1). We will provide early preclinical and clinical evidence that tumor and immune responses to paclitaxel released from locally administered SPP are different than responses to paclitaxel delivered intravenously (IV). These findings support the conclusion that SPP may offer an important addition to cancer therapy without adding clinically significant local or systemic toxicity.

**Table 1:** Clinical trials of NanoPac®

Study ID	NCT number	Study title	Study start	Study status^a^
HSC#1114	NCT00666991	Pharmacokinetic, safety and efficacy study of nanoparticle paclitaxel in patients with peritoneal cancers	July 2008	Completed
NANOPAC-2016-01	NCT03029585	Phase II study of four dose levels of intraperitoneal NanoPac Plus IV carboplatin and paclitaxel in patients with epithelial ovarian cancer undergoing cytoreductive surgery	April 2017	Completed
NANOPAC-2016-02	NCT03077659	Phase IIa dose escalation trial of NanoPac focal therapy for prostate cancer in subjects undergoing radical prostatectomy	September 2017	Completed
NANOPAC-2017-01	NCT03188991	A trial evaluating escalating doses and the safety of intracystic injection of NanoPac in subjects with mucinous cystic pancreatic neoplasms	September 2017	Completed
NANOPAC-2016-05	NCT03077685	Phase IIa trial evaluating the safety of intratumoral injection of NanoPac in subjects with locally advanced pancreatic adenocarcinoma	December 2017	Ongoing
NANOPAC-2019-01	NCT04221828	Phase 2 trial of NanoPac focal therapy for prostate cancer in subjects undergoing radical prostatectomy	July 2020	Recruiting
NANOPAC-2020-01	NCT04314895	Phase 2 trial evaluating the safety and tolerability of intratumoral injections of NanoPac® with standard of care therapy in subjects with lung cancer	September 2020	Recruiting

^a^ = Study status current as of 5 October 2020

## Preparation of submicron taxane particles

**Fig. 1:**
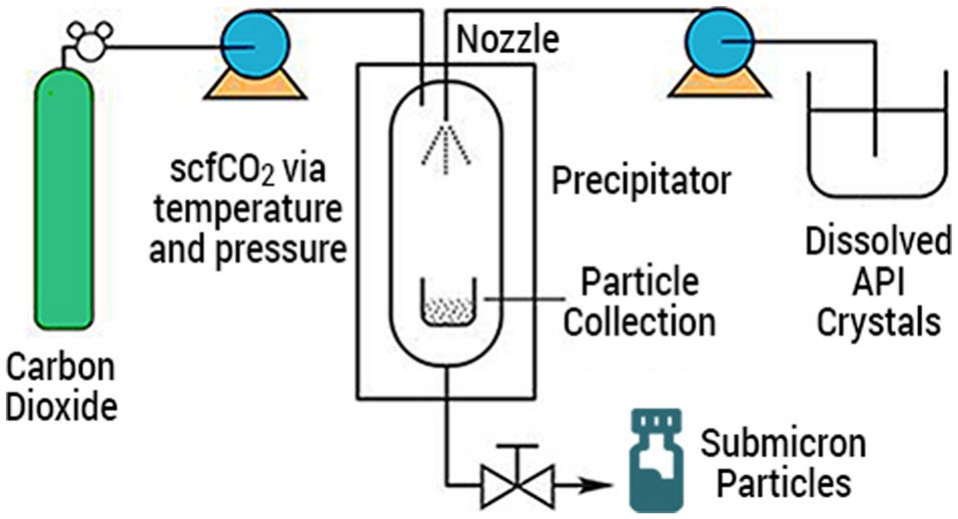
Schematic of continuous manufacturing process for submicron particle paclitaxel. Supercritical carbon dioxide (> 72.8 bar, > 31 °C) (ScCO_2_) is freely soluble with many organic solvents but insoluble with paclitaxel. Paclitaxel dissolved in organic solvent is sonicated into small droplets and rapidly exposed to scCO_2_, which strips away the solvent and precipitates the paclitaxel as small particles. These small, stable, flowable particles are captured in a collection chamber and later filled in powder form into vials

**Fig. 2:**
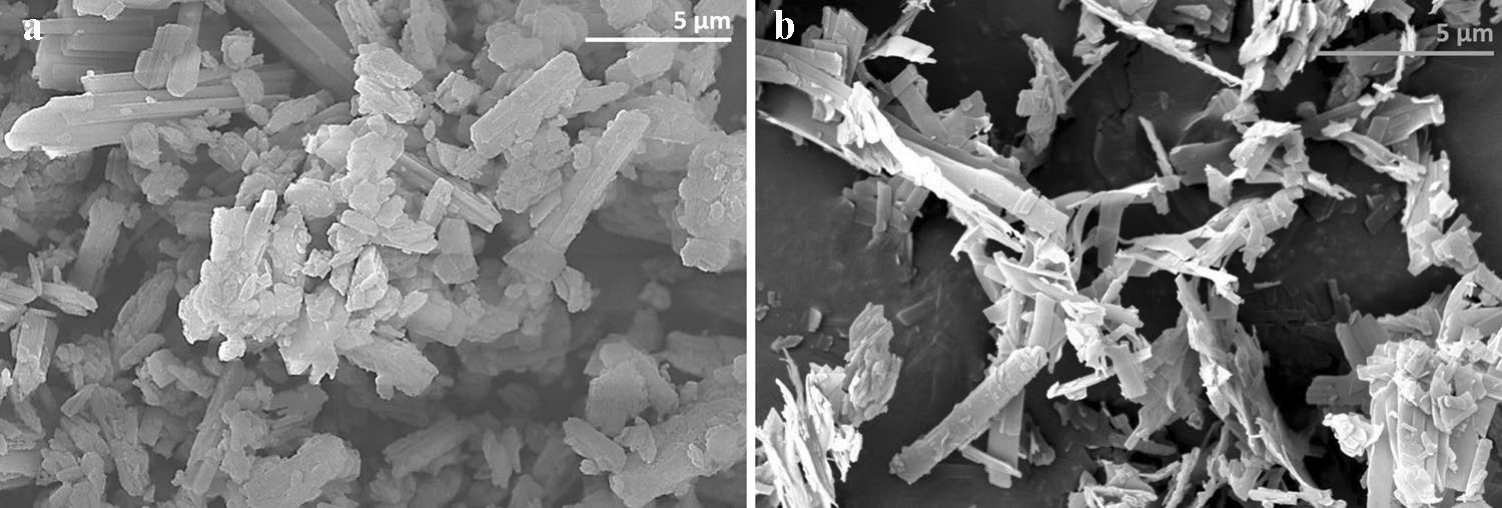
(**a**) Paclitaxel drug substance and (**b**) Paclitaxel drug substance precipitated with compressed antisolvents results in particles of paclitaxel with a specific surface area > 22 m^2^/gm with a bulk density between 0.05 and 0.15 gm/cm^3^

Precipitation with compressed antisolvents (PCA) is a non-solvent process by which a drug substance dissolved in an organic solvent is precipitated in a supercritical antisolvent. Baltezor et al. [5] described a method by which paclitaxel dissolved in acetone processed with supercritical CO_2_ created SPP with a number-weighted mean particle size ranging from 0.670 to 0.861 μm with a specific surface area greater than 22 m^2^/g and a bulk density between 0.05 and 0.15 g/cm^3^ (Fig. 1; CritiTech, Lawrence, KS). The volume-weighted mean particle size distribution for this product ranged from 2.8 to 3.5 μm and matches the size of the particles found by scanning electron microscopy (SEM). Figure 2 a and b is SEM photomicrographs of the unprocessed and PCA processed paclitaxel. They reveal morphological differences between unprocessed and PCA-processed particles. Unprocessed paclitaxel crystals were rod-shaped, thicker, and clumped together resulting in a large size variability. The PCA-processed SPP were much thinner, highly irregular shaped particles with large open areas between particles.

**Fig. 3:**
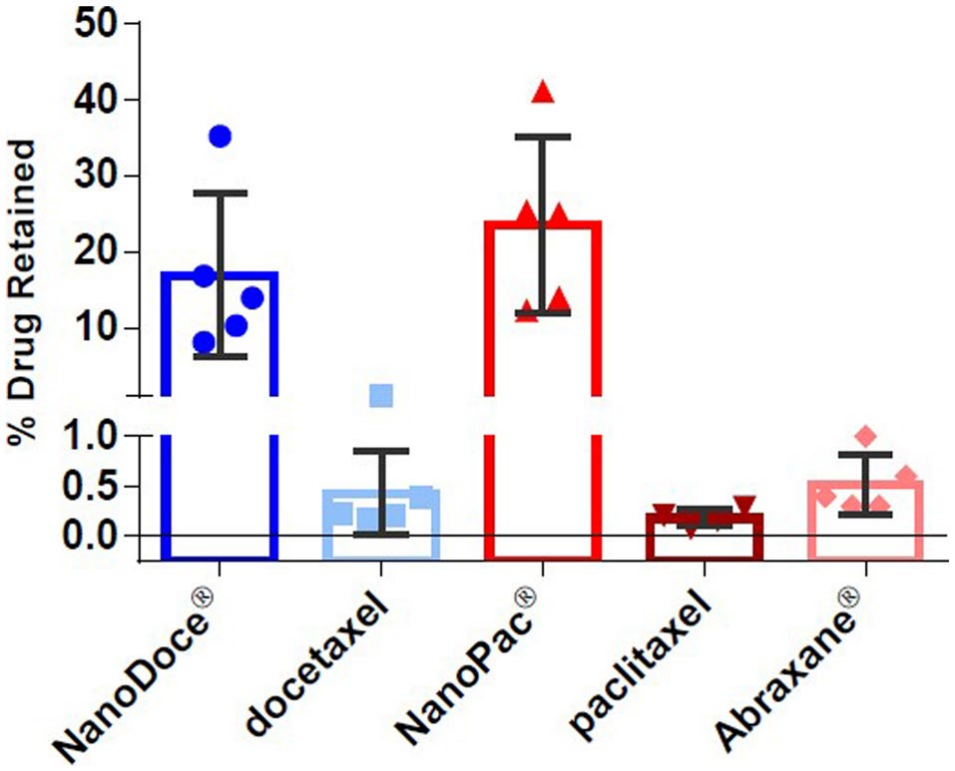
Percent drug retained in MDA-MB-231 tumors. Formulations (5 mg/mL each) were administered as 0.05 mL direct injections into MDA-MB-231 tumors implanted on the flanks of female BALB/c mice (*n* = 4 or 5 per group), and tumor tissues were collected 5 days later. The percent of drug retained was calculated based on the concentration of drug detected (ng/g of tumor) and tumor weight. Group mean docetaxel (blue bars) and paclitaxel percent (red bars) as well as individual tumor percent (symbols) are plotted; error bars = ± 1 SD

The SPP particles had an increased specific surface area (SSA) which is equivalent to much smaller particles. The increased SSA enhances the release rate of drug from the particles. The irregular shape of the particles is thought to result in interlocking between particles, causing them to function as if they were even larger. We theorize these particles are large enough to avoid being removed by blood flow or by phagocytosis and are retained in tumors, creating a sustained drug release depot while minimally contributing to systemic paclitaxel levels. Parallel PCA processes can also be employed to generate submicron particle docetaxel (SPD). When administered to tumors as an aqueous suspension, the PCA particles release paclitaxel [6, 7] or docetaxel [8] into the surrounding tumor microenvironment (TME) at tumoricidal levels (Fig. 3).

These large agglomerated particles with the high SSA and the low bulk density are also excellent candidates for enhancing pulmonary drug delivery. The mass median aerodynamic diameter (MMAD) of PCA particles is typically in the range of 2 to 4 μm. However, the physical size of the particles or agglomerates of the particles is > 5 μm which is large enough to inhibit removal from the lungs by phagocytosis. We have observed that PCA-produced particles provide much longer lung residence times [6].

The PCA process is adjustable to allow for particle engineering of a specific drug to create particles with the most desirable characteristics for an intended delivery method. Processing variables of the solvent, the concentration of drug in the solvent, the spray rate of the drug solution, the intensity of the sonic energy as well as the flow rate, and pressure of the supercritical CO_2_ are examples. Once defined and controlled, the PCA process is reproducible with high yields (> 95%) and low residual solvent.

Prior to the start of the Phase 2 clinical trials, 3 batches of SPP were manufactured under full GMP conditions using continuous process of approximately 36 h each. The batches shown in Table 2 were used for the Phase 2 clinical supplies and for providing ICH compliant stability data for SPP. Table 2 shows the results at *T*_0_ and after 48 months storage at 25°C 60%RH in 60 ml sterile glass vials. The data demonstrates the consistency and excellent stability of the 3 batches. The batches have been monitored with Xray powder diffraction (XPRD) and differential scanning calorimetry (DSC) to confirm that there have been no changes in the crystalline form of the particles. All three batches had an untapped bulk density of ~ 0.08 g/cm^3^. The particle size by volume (Dv50) shown in Table 2 was found to be a critical parameter in the understanding of the relationship between the particle size, the bulk density and the high specific surface area of the particles. We are still in the process of finalizing an in vitro dissolution test, but we have found that the specific surface area of the particles has a direct linear correlation with the dissolution results for the particles.

**Table 2:** Results of 3 GMP NanoPac® batches used for Phase 2 clinical studies and ICH compliant stability

NanoPac®	Assay (%)		Particle size (Dn50) (*µ*)		Particle size (Dv50) (*µ*)	Surface area (m^2^/g)	
Lot number	*T*_0_	48 months	*T*_0_	48 months	48 months	*T*_0_	48 months
STV090915	100.4	98.2	0.79	0.89	2.86	29.9	28.4
STV093015	98.2	98.2	0.76	0.89	3.22	29.1	26.1
STV100715	98	98	0.83	0.85	3.2	27.9	25.9

## Preclinical studies

### Lung cancer

**Fig. 4:**
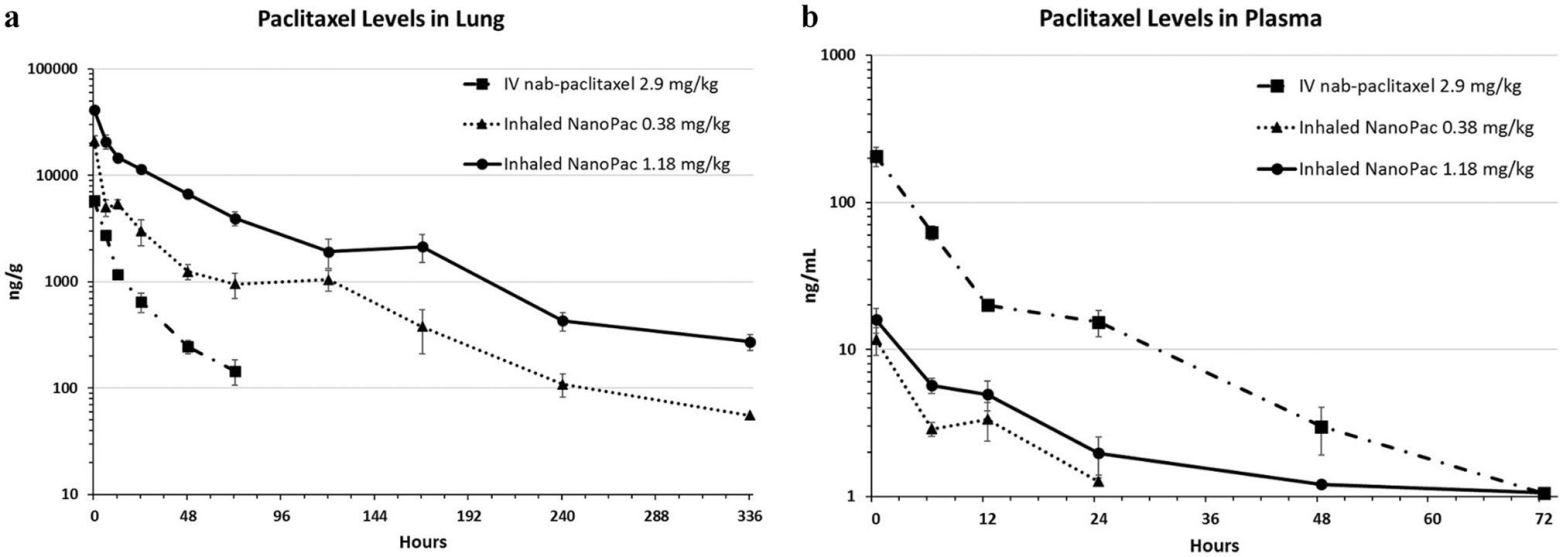
Paclitaxel levels in lung (**a**) and plasma (**b**) after inhaled NanoPac treatment. Male Sprague–Dawley rats (6–8 weeks old) were administered paclitaxel on a single occasion in one of three treatment arms (*n* = 30 each): inhaled submicron particle paclitaxel in a nose-only exposure at a low-dose of 0.38 mg/kg or a high dose of 1.18 mg/kg, or intravenous nab-paclitaxel administered via tail vein injection at 2.9 mg/kg. Three animals from each arm were sacrificed at 0.5, 6, 12, 24, 48, 72, 120, 168, 240, and 336 h post exposure for lung tissue and plasma collections. Lung tissue (**a**) and plasma (**b**) were assayed via ultra-performance liquid chromatography tandem mass spectrometry to quantify paclitaxel concentration as a function of time with a lower level of quantification of 50 ng/g and 1 ng/mL, respectively (mean ± 1SEM) [6]. Reprinted by permission from Mary Ann Liebert, Inc.: [Mary Ann Liebert] [Journal of Aersol Medicine and Pulmonary] [Pharmacokinetic profile of inhaled submicron particle paclitaxel (NanoPac) in a rodent model, James Verco et al.] [2019]

Efficacy of IV paclitaxel in the treatment of lung cancer is limited by its concentration and duration of tumor exposure. Alternate routes of administration have been evaluated [9] including IH and IT injections by bronchoscopy [10]. Studies of IH paclitaxel demonstrated preliminary proof-of-efficacy in non-small cell lung cancer (NSCLC) preclinical models [11]. Inhaled chemotherapy could theoretically deliver substantial doses directly to the pulmonary parenchyma and adjoining airways while avoiding additive toxic exposure to nontarget organs. Historically, however, achieving sustained pulmonary exposure through IH was limited by poor retention of drug within the pulmonary tissues due to clearance mechanisms such as diffusion across the alveolar–capillary membranes, the mucociliary “escalator” removing material to the gastrointestinal tract, and phagocytosis by alveolar macrophages and dendritic cells, as well as lymphatic drainage. To address these challenges, IH of nebulized SPP was evaluated. Following IH of nebulized SPP, substantial levels of paclitaxel in the lung were achieved for at least 2-week post-administration (Fig. 4), confirming the increased local retention and efficacy of IH SPP [6, 7].

**Fig. 5:**
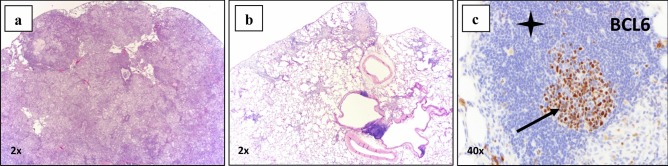
In an orthotopic nude NIH male rat lung cancer model (Calu-3) was treated with inhaled submicron particle paclitaxel twice weekly for 4 weeks at 0.5 and 1.0 mg/kg. All animals survived to their scheduled necropsy and exhibited no adverse clinical observations from treatment. Left lungs were paraffin embedded, serially sectioned and stained for histopathological and immunohistochemical examination. (**a**) Control group treated with vehicle exhibited unabated tumor growth; (**b**) inhaled submicron particle paclitaxel groups presented a reduced or absent tumor presence and contained occasional areas of residual fibrosis; (**c)** BCL-6 stained follicles revealed localized B-cells at the germinal center (black arrow) and B-cells in the submicron particle inhaled groups (black star) which reside in the surrounding mantle zone [7]. Reprinted by permission from Mary Ann Liebert, Inc.: [Mary Ann Liebert] [Journal of Aersol Medicine and Pulmonary Delivery] [Inhaled submicron particle paclitaxel (NanoPac) induces tumor regression and immune cell infiltration in an orthotopic athymic nude rat model of non-small cell lung cancer, James Verco et al.] [2019]

In NIH-nru nude rats orthotopically implanted with Calu-3 cells (human lung adenocarcinoma) treatment with inhaled SPP resulted in tumor growth inhibition, regression, and infiltration [7]. In some lung samples, no residual tumor was detected upon histopathological examination; scattered, small fibrotic nodules, and stroma replacing areas presumed to have once contained tumor was observed. Tumor regression and eradication were accompanied by a robust immune cell infiltrate of lymphocytes and macrophages into the tumor spaces. Since these animals were athymic and thus deficient in T cells, any immune-mediated tumoricidal affect would have been due to macrophage infiltration and primarily antibody-dependent, cell-mediated cytotoxicity (ADCC) or antibody dependent, and cell-mediated phagocytosis (ADCP). Paclitaxel is known to enhance ADCC [12]. These two anti-tumor responses depend on both the presence of antibodies against the tumor and the presence of immune effector cells. Immunohistochemical staining (IHC) of lung tissue showed an increase in CD11b + immune cell infiltration in lung samples following IH SPP. Some of the bronchus associated lymphoid tissue (BALT) exhibited architecture consistent with active germinal centers within lymphoid follicles (Fig. 5). These findings suggest that persistent paclitaxel release at high concentrations facilitates antigen presentation to the host immune system and that antigen availability enhances immune cell infiltration into tumor sites.

### Genitourinary neoplasms

**Fig. 6:**
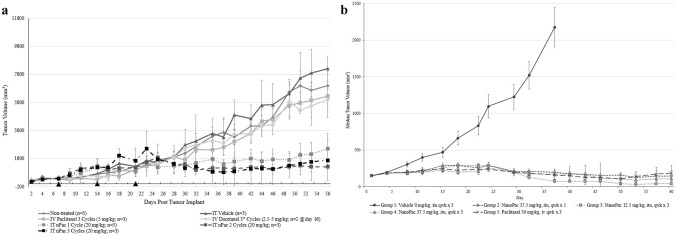
(**a**) Mean tumor volume in 786-O renal cancer xenograft in 5–7-week-old female Sprague–Dawley *Rag2; Il2rg (null)* rats (*n* = 2–3 animals/group) following IT treatment with submicron particle paclitaxel (nPac; 20 mg/kg). Treatments were initiated seven days after tumor implant and administered weekly for one, two or three cycles (black triangles). Control groups included no treatment, IT vehicle administered on the same schedule as IT submicron particle paclitaxel, IV paclitaxel, or IV docetaxel. * = due to toxicity, following the first cycle the IV docetaxel regimen was modified from 5 to 2.5 mg/kg for one or two additional cycles. Tumors were measured with calipers three times weekly for the duration of the study [13]. (**b**) Median tumor volume in PC-3 prostate cancer xenograft in 11-week-old female NCr nu/nu mice (Crl:NU(NCr)-Foxn1^nu^; *n* = 10 animals/group) following IT treatment with submicron particle paclitaxel (NanoPac; 12.5 to 37.5 mg/kg) for 1 or 3 weekly cycles. Control groups included IT vehicle or IV paclitaxel. Treatments were initiated 35 days after tumor implant on Day 1 of the study. Tumors were measured with calipers twice weekly for the duration of the study. [14]. Error bars ± 1 SDEV

Direct IT injection of SPP into xenograft models of human prostate and renal cell carcinoma lines produced regression or eradication of tumors (Fig. 6a,b). Formation of fibrin deposits, presumably from tumor debris, was associated with a robust immune cell infiltrate at the tumor site. In contrast, animals that received IV taxane showed continued tumor growth and modest to no immune cell infiltration [8, 13, 14]. These observations suggest that the response of carcinomas to prolonged exposure to tumoricidal levels of taxanes may involve at least two different mechanisms. First, direct tumor kill by the taxane-induced disruption of mitosis is followed by tumor cell disruption, making neoantigens available and causing antigen spread within the TME. Second, these conditions stimulate immune effector cells to further the local tumoricidal response.

## Clinical trials

### Intraperitoneal submicron particle paclitaxel

**Fig. 7:**
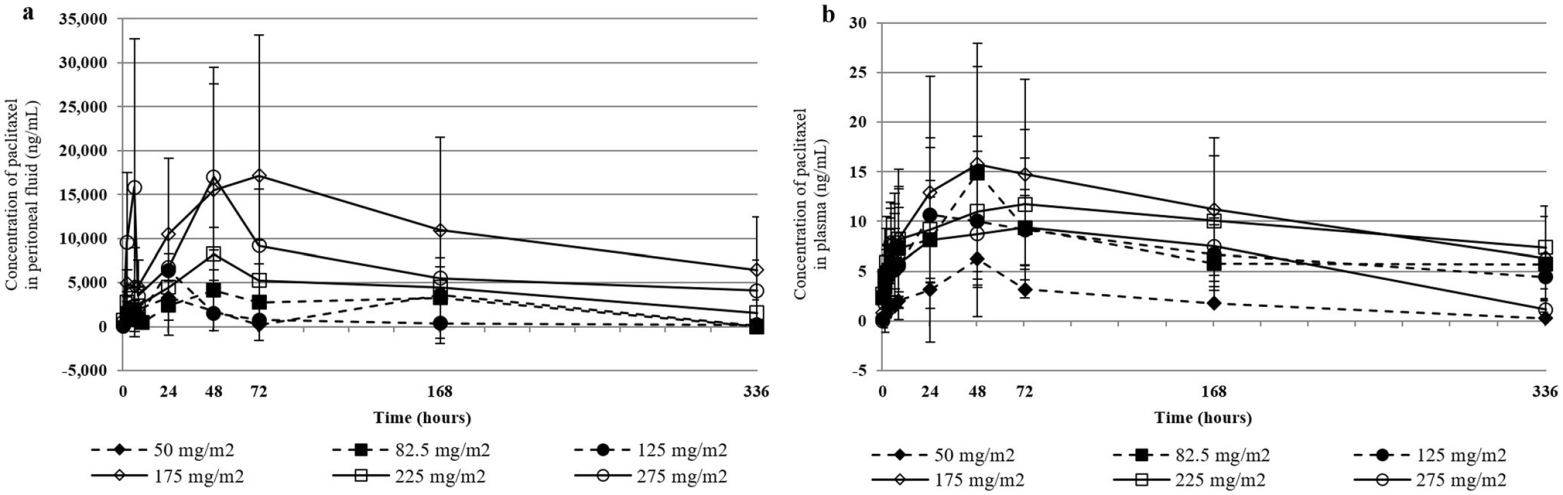
Peritoneal fluid (**a**) and plasma (**b**) paclitaxel concentrations following intraperitoneal administration of submicron particle paclitaxel. Data are averaged over cycles 1 and 2 in subjects with intraperitoneal carcinomas, mostly recurrent ovarian cancer. Mean peritoneal fluid and mean plasma concentrations are presented per dose level. Error bars ± 1 SDEV [15]. Reprinted by permission from Springer Nature: [Springer] [Cancer Chemotherapy and Pharmacology] [A phase I study of intraperitoneal nanoparticulate paclitaxel (Nanotax®) in patients with peritoneal malignancies, Stephen K. Williamson et al.] [2015]

The first clinical study of SPP investigated IP administration in patients with carcinoma predominantly confined to the peritoneal cavity who failed prior therapies (NCT00666991, [15]). Twenty-one subjects received SPP via IP ports in doses ranging from 50 to 275 mg/m^2^. These subjects previously underwent treatments, including IV chemotherapy and debulking surgery. Treatment cycles with IP SPP were 28 days long with one to six cycles administered with the majority of subjects received two cycles. IP administration of SPP did not induce peritonitis nor systemic toxicity typically associated with IV paclitaxel. The peritoneal levels of paclitaxel rose during the 2 days after dosing to concentrations 450–2900 times the peak plasma paclitaxel concentrations and remained elevated throughout the treatment cycle (Fig. 7). Plasma paclitaxel levels remained below 10 ng/mL, well below the systemic toxicity threshold of 40 ng/mL [16]. Objective tumor response assessed by RECIST 1.0 occurred in five subjects with stable disease and 15 with progressive disease. Six of 21 subjects survived ≥ 1 year, and three survived ≥ 2 years despite their advanced disease. Compared with IP administration of nab-paclitaxel in a separate study [17], IP administration of SPP provided higher, sustained peritoneal paclitaxel levels with minimal systemic exposure and reduced toxicity [15].

A Phase 2 study in 10 subjects with primary or recurrent ovarian cancer was subsequently conducted to evaluate a single dose of SPP instilled into the peritoneal cavity at the end of debulking surgery (NCT03029585). Subjects also received standard-of-care (SOC), which consisted of IV paclitaxel and carboplatin administered once every 21 days for up to 6 cycles. In the study, 66% of subjects who received SOC plus a single dose of SPP showed progression-free survival (PFS) over the 12-month study [18].

PK sampling was conducted in seven subjects who received SPP at a dose of 100 mg/m^2^ and three subjects who received a dose of 200 mg/m^2^. Blood samples were collected immediately following SPP instillation, prior to each cycle of IV chemotherapy, and at 9- and 12-months post-administration. Plasma paclitaxel concentrations in the 100 mg/m^2^ dose group were quantifiable (> 25 pg/mL) in three of seven subjects prior to Cycle 6 of IV chemotherapy. Quantifiable plasma paclitaxel concentrations were detected in two of three subjects in the 200 mg/m^2^ dose group prior to the Cycle 3 of IV chemotherapy and in 1 subject at 12 months following SPP instillation. Given that the reported half-life for IV paclitaxel ranges from 9.9 to 16 h for a 3-h infusion and 13.1 to 24.6 h for a 24-h infusion [19], paclitaxel would have cleared systemic circulation by the sixth day following IV administration and thus, not be quantifiable prior to the next cycle of IV paclitaxel. Since plasma paclitaxel concentrations were quantifiable just prior to the 21-day dosing cycle of IV paclitaxel and at the 9- and 12-month follow-up visits, it is likely that SPP acted like a paclitaxel-depot that released drug for an extended period following IP administration. These studies support further evaluation of SPP administered into the peritoneal cavity to treat IP carcinomas.

### Submicron particle paclitaxel intratumoral injection

#### Prostate cancer

Subjects (*n* = 16) with adenocarcinoma of the prostate and a Gleason score ≥ 7 were treated via transrectal ultrasound (TRUS)-guided injection of SPP into the index tumor and its lobe that contained the index tumor using multi-parametric magnetic resonance imaging (mpMRI) guidance (NCT03077659). Subjects were allocated to cohorts by a standard 3 + 3 dose rising design to receive 6, 10, or 15 mg/mL SPP in a volume equal to 20% of the lobe containing the index lesion, with additional subjects enrolled to an expansion cohort at the 15 mg/mL dose. A radical prostatectomy was performed 4 weeks after IT SPP treatment. There were no drug-related serious adverse events (SAEs), including no prostatitis, nor dose-limiting toxicities (DLTs), allowing for dose-escalation to the highest concentration (15 mg/mL). Paclitaxel was detected in all prostate tissue sampled from prostatectomy specimens and in lymph nodes of nine subjects > 30 days following intraprostatic (ITP) SPP treatment. Percentage of adenocarcinoma in core biopsies decreased between screening and prostatectomy in six subjects and remained unchanged in five. Among seven subjects that received 15 mg/mL, the mean lesion volume (as measured by mpMRI) decreased, as did the prostate specific antigen (PSA) density. ITP SPP treatment was found to be well tolerated with no safety concerns at the 15 mg/mL concentration and provided preliminary evidence of activity.

A second clinical trial is underway in patients with localized prostate cancer (Gleason ≥ 6) utilizing multiple injections over a 16-week period prior to prostatectomy (NCT04221828) to further evaluate safety, efficacy, and immune response. The lack of systemic toxicity and minimal local irritation observed following ITP SPP treatment in the first prostate cancer study may allow for multiple staged injections in future studies, the timing of which could be triggered by rising PSA levels during routine SOC. Persistent paclitaxel within the prostate may act as an adjuvant “sensitizer” to radiation therapy or focal ablative therapies such as high-intensity focused ultrasound (HIFU).

#### Pancreatic cancer

Pancreatic cancer demonstrates a limited response to systemic cancer therapy. This may be due in part to the aggressive nature of tumor cells and to the desmoplastic, fibrotic stroma associated with pancreatic cancer, which blocks transport and diffusion of small molecules [20, 21]. The dense stroma also creates damaged or “leaky” blood vessels causing poor drainage of lymphatic and vascular fluids. Excess fluid increases interstitial fluid pressures, thus compressing vessels and decreasing micro-vessel density [20, 22]. The poorly vascularized stroma creates a hypoxic environment, suppressing the immune response and forming a barrier to systemic drug delivery. Surgical resection of the tumor, when possible, is currently the only curative therapy. Unfortunately, surgical resection is often not possible due to anatomical complications when the patient initially presents for medical care.

IT SPP via endoscopic ultrasound-guided fine needle injection (EUS-FNI) has shown promise in a clinical trial of patients with locally advanced pancreatic cancer (LAPC) (NCT03077685, [23]). Subjects with non-resectable LAPC lesions with a diameter of 1.5–6 cm were directly injected with SPP via EUS-FNI in a 3 + 3 dose-escalation design at 6, 10, and 15 mg/mL in volumes up to 20% of the tumor (not exceeding 5 mL). Subjects in the second phase of the study received two SPP injections 4 weeks apart at 15 mg/mL. An additional cohort of subjects is now open to recruitment, where up to four injections will be given via EUS-FNI one month apart. Injections are performed through a 22-gauge needle in a fan-like pattern to ensure drug dispersion throughout the tumor.

In the 29 subjects to date who have received one (*n* = 7) or 2 (*n* = 22) injections of SPP at 15 mg/ml, there have been no cases of pancreatitis, no SAEs definitely related to drug, and no clinically significant laboratory abnormalities. There have been no adverse events that were considered to be dose-limiting. Paclitaxel in the plasma was detected at levels below 10 ng/mL during the first 24 h after IT SPP administration, returning to undetectable levels by 4 weeks [23, 24].

**Fig. 8:**
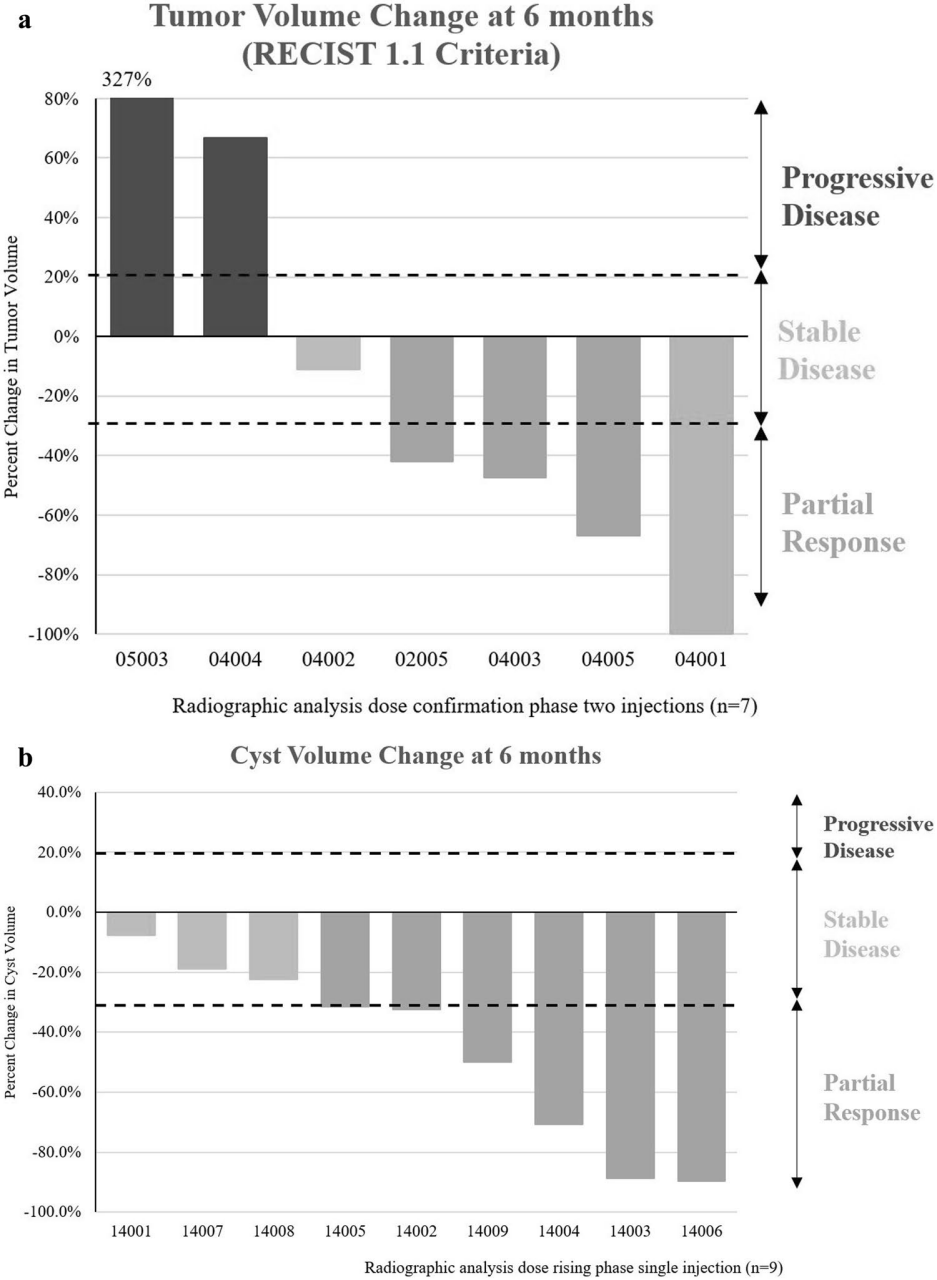
Change in tumor volume of locally advanced pancreatic cancer following two IT injections of submicron particle paclitaxel (**a**) and mucinous pancreatic cysts in subjects following a single injection of submicron particle paclitaxel via EUS-FNI (**b**). No subject developed clinically significant local or systemic toxicity from the drug or injection(s) [23, 25]

RECIST 1.1 evaluation of the tumor volumes in the two-injection group of subjects demonstrated stable disease in two of eleven subjects at 6 months (Fig. 8a), progressive disease in two subjects, and partial or full responses in seven subjects. Thus far, one subject (04001) in the second phase who received 2 injections was down-staged during study and underwent surgical resection of tumor resulting in R0 and LN0 outcomes [23], and other subjects are under evaluation for reassessment of surgical status.

#### Pancreatic mucinous cysts

Pancreatic mucinous cystic neoplasms (MCNs) and intraductal papillary mucinous neoplasms (IPMNs) have significant potential to undergo malignant transformation into pancreatic cancer [26]. These cysts are at high risk for progression and often require precautionary pancreatectomy, a procedure associated with high morbidity and potential for cyst recurrence. Ethanol ablation followed by paclitaxel administration via EUS-FNI is a minimally invasive technique that has shown benefit in the treatment of cystic lesions [27, 28].

A trial evaluating intracystic administration of SPP by EUS-FNI into mucinous pancreatic cysts has completed (NCT03188991). This trial was similar in design and dose to the pancreatic cancer trial, with a dose escalation phase (SPP at 6, 10, and 15 mg/mL at volumes sufficient to fill the cyst, at least equal to the amount of cyst fluid aspirated) followed by a second phase in which subjects received two administrations of SPP at 15 mg/mL administered 12 weeks apart. Patients were followed for 6 months after the first injection. Nineteen patients were enrolled in the study. Nine patients have completed the dose-rising phase of the study and eight have completed the second (two-injection) phase. No clinically significant local toxicity or significant laboratory abnormalities from SPP have been observed. Plasma paclitaxel concentration did not exceed 3.5 ng/mL at any timepoint measured and fell below 1 ng/mL by Week 2, supporting retention of paclitaxel particles within the pancreatic cyst [25]. Currently, cyst volumes in eight of the nine evaluable subjects in the dose escalation cohorts remain reduced at Month 6 (Fig. 8b).

## Mechanism of action

Paclitaxel’s primary cytotoxic mechanism acts by inhibiting tubulin depolymerization, stalling the cell cycle in the G2/M phase, interfering with tumor cell replication and resulting in tumor cell death [29] which can produce both apoptotic as well as necroptotic cell death. Necroptosis (necrosis) is a drug-and dose-dependent mechanism of tumor cell destruction achieved by the persistence of relatively high levels of chemotherapy in the TME. In contrast to apoptosis, which often includes collapse and contraction of tumor cells, necroptosis is associated with loss of tumor cell membrane integrity, exposing tumor-specific antigens to immune surveillance. This process can stimulate a robust response of the adaptive immune system against the tumor-specific antigens that otherwise would remain “unseen” by immune effector cells, enhancing immune effector cell infiltration into the TME [30–32]. Paclitaxel was shown to induce necroptotic tumor cell death following exposure to high drug concentrations [33]. The histologic patterns of tumor cell death following local administration of SPP are reminiscent of those associated with necroptotic tumor cell death [7, 30]. Thus, SPP-induced cell death appears to be a continuum between apoptosis and necroptosis depending on drug concentration and duration of tumor cell exposure [31, 34, 35].

## Summary and conclusions

Local administration of SPP continuously exposes primary tumors to therapeutic levels of paclitaxel for several weeks. Continuous exposure of solid carcinomas to tumoricidal levels of paclitaxel was shown in preclinical studies and early, limited clinical trials to provide clinical benefits with minimal local or systemic toxicity. Paclitaxel-induced neutropenia and other systemic toxicities are related to the duration and extent of systemic exposure to paclitaxel above a threshold plasma concentration of ≥ 40 ng/mL [16]. In contrast, plasma paclitaxel concentrations observed after injecting SPP into the lobe of the prostate had *C*_max_ values of 19 to 20 ng/mL recorded at the 1-h timepoint. By comparison, a standard IV dose of paclitaxel of 175 mg/m^2^ administered over 3 h results in a *C*_max_ of 3,650 ng/mL. This is approximately 192 × higher than the mean paclitaxel concentration following SPP injection in a dose of 15 mg/ml in a volume 20% of the prostate lobe 1 h after injection. In addition, measurements of plasma paclitaxel levels following IT SPP in pancreatic cancer trials and IP SPP in peritoneal cancer trials remained well below the 40 ng/mL toxicity threshold. These low levels of plasma paclitaxel following local administration of SPP may explain the absence of clinically significant toxicity seen thus far in various clinical trials (Table 1) involving more than 150 subjects.

SPP administration may complement treatment of metastatic disease with traditional therapies such as chemotherapy, targeted therapy, immunotherapy, and radiation. Paclitaxel affects many aspects of immune function, including lymphocyte recruitment and activation as well as production of immunoenhancing cytokines, including IL-12 [34–37], IFNγ and TNFα, which may augment the antitumor activity of immunotherapies [38]. Paclitaxel was shown to enhance immune responses including increased concentrations of tumor-infiltrating lymphocytes that successfully eradicate malignant cells [39–41]. Paclitaxel may be a particularly strong immunostimulant, as it is able to both activate CD8 + T cells and reduce immunosuppressive cells, such as regulatory T cells [34–36, 42–44] and myeloid-derived suppressor cells (MDSC) [30, 36, 45]. To maximize tumoricidal effects, it has been hypothesized that the immune system can be primed with systemic chemotherapy ahead of immunotherapy to re-instate or enhance immunosurveillance [37, 39]. The priming effect of locally administered SPP may not only provide tumoricidal activity but also induce immune-mediated effects [37, 46–49]. The persistence of paclitaxel in the tumor site may expose slowly replicating tumor cells to tumoricidal drug levels. This chronic state of cell death could affect the immune system by (1) increasing the opportunity for tumor-associated antigens to be released and recognized by the immune system, (2) decreasing the number of cells able to send an immunosuppressive signal, and (3) attracting immune and phagocytic cells to remove tumor cell debris. While a combination of systemic chemotherapy and immunotherapy has the potential to increase efficacy [46], these regimens are also additive to systemic toxicity. Local administration of SPP has the potential to synergize with immunotherapy without added toxic exposure to nontarget organs.

Injection of SPP into a primary tumor appears to facilitate an immune response which may improve clinical benefits reminiscent of those reported in studies where radiation and ablation were used to create an “inflamed” TME that synergizes with immune therapy [47–56]. Preliminary studies of SPD injected into syngeneic Renca tumors also produced reduction in tumor size with associated increases in effector immune cell concentrations [57]. Potential therapeutic opportunities for administration of SPP to treat carcinomas include (1) inhalation of nebulized particles and/or direct injection of tumors obstructing airways to treat pulmonary cancers; 2) IP SPP to treat peritoneal metastasis, ovarian, hepatic, and other gastrointestinal cancers; (3) direct injection via TRUS-FNI to achieve reduction of prostate cancer or delay progression, allowing for delay or prevention of prostatectomy or whole gland therapy; (4) direct injection via EUS-FNI into pancreatic cancer; and (5) intracystic injection of pancreatic mucinous cysts to prevent partial pancreatectomy. The broad antitumor effects and safety observed preclinically and clinically, and the apparent stimulation of the immune system following SPP treatment support additional preclinical and clinical investigations with this drug.

### Open access

This article is distributed under the terms of the Creative Commons Attribution 4.0 International License (https://creativecommons.org/licenses/by/4.0/), which permits unrestricted use, distribution, and reproduction in any medium, provided you give appropriate credit to the original author(s) and the source, provide a link to the Creative Commons license, and indicate if changes were made.

## Data Availability

The datasets generated during and/or analyzed during the current study are available from the corresponding author on reasonable request.
